# T-cell repertoire correlates with cytokine imbalance in multiple sclerosis patients

**DOI:** 10.3389/fimmu.2025.1604452

**Published:** 2025-07-01

**Authors:** Lisa Weidner, Rodolphe Poupardin, Tobias Zrzavy, Sandra Laner-Plamberger, Georg Gratz, Tanja Eichhorn, Viktoria Weber, Paulus S. Rommer, Christof Jungbauer, Dirk Strunk

**Affiliations:** ^1^ Blood Service for Vienna, Lower Austria and Burgenland, Austrian Red Cross, Vienna, Austria; ^2^ Department for Transfusion Medicine, University Hospital (SALK), Paracelsus Medical University (PMU), Salzburg, Austria; ^3^ Cell Therapy Institute, Paracelsus Medical University, Salzburg, Austria; ^4^ Department of Neurology, Medical University of Vienna, Vienna, Austria; ^5^ Department for Biomedical Research, Center for Biomedical Technology, University for Continuing Education Krems, Krems, Austria

**Keywords:** neuroscience, multiple sclerosis, T cell repertoire, cytokine imbalance, bioinformatics

## Abstract

**Indroduction:**

Multiple sclerosis (MS) is mediated by innate and adaptive immune response deviation involving immune cells and cytokines. Here, we investigated whether combined cytokine profiling and T-cell receptor (TCR) repertoire analysis can better display the complex landscape of MS-driving immune responses.

**Methods:**

We used advanced computational methods to systematically cluster highly variable individual levels of 48 cytokines in cerebrospinal fluid (CSF) and blood of 24 MS patients compared to that of nine controls. Relevant TCR sequences were compared to 88 healthy controls. We correlated cytokines with predominant shared TCR sequences to identify immune response networks.

**Results:**

MS patients had significantly elevated MIP-1α and IP-10 levels in CSF, and additional 36 blood cytokines variably but significantly elevated. We identified 77 predominantly pro-inflammatory cytokine correlations in MS-CSF. TCR sequencing revealed more productive rearrangements in CSF of MS and a significantly higher shared clone recovery rate in blood. We found significant associations involving 492 unique sequences and 34 cytokines in blood. Particularly, the less significant individual cytokine deviations were found to contribute to a general Th1-biased type I immune response correlating with clonal expansion of T cells directed against EBV, CMV, and other infectious agents.

**Discussion:**

Correlation of significantly altered T-cell repertoire with cytokine deviations in MS despite individual patient data variability indicates that future diagnostic strategies may need to address immune response patterns rather than individual protein targets.

## Graphical Abstract

Correlation of cytokine profile and T-cell receptor (TCR) repertoire in cerebrospinal fluid (CSF) and blood of multiple sclerosis (MS) patients compared to that in control patients. Differential expression in MS (arrow up for IP-10 and MIP-1α) and selected significant correlation pattern (adjusted p < 0.05) found between different cytokines (color-coded rings): positive (pink) and negative correlations (light blue). The integrative analysis in this study revealed a pro-inflammatory cytokine signature, predominantly driven by type I immunity and Th1-related cytokines (e.g., IFN-γ and TNF-β), with significant correlation between specific TCR sequences (directed against infectious targets) and cytokine levels, primarily in blood, with just limited overlap to CSF. Cytokine-TCR interactions are considered to extend across the altered blood-brain barrier.

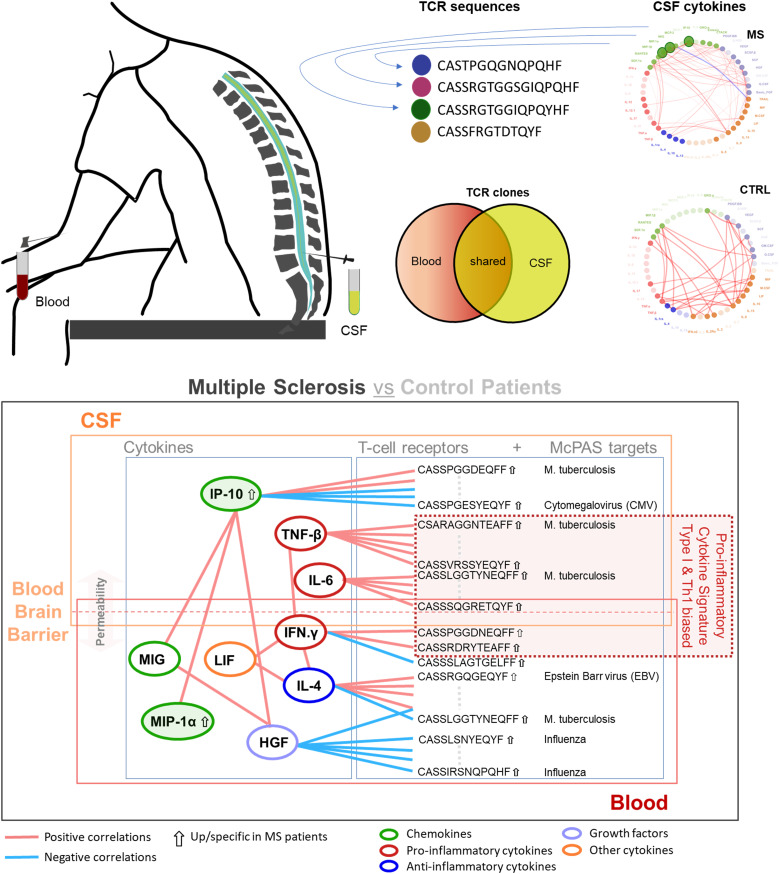

## 1 Introduction

Multiple sclerosis (MS) is a chronic demyelinating autoimmune disease, which commonly clinically manifests with acute bouts of neurological deficits followed by intervals of apparent steadiness. The development of the disease is influenced by genetic susceptibility combined with environmental immune triggers (1, 2). The role of Epstein–Barr virus (EBV) infection in the development of MS has most recently been strengthened, but it is not yet clear how exactly EBV contributes to the manifestation of MS (3, 4). The hypothetic mechanistic basis of relapses relates to autoreactive T cells that invade the central nervous system and trigger an inflammatory cascade, thus damaging the myelin sheath of axons, leading to axonal loss and apoptosis of oligodendrocytes (2).

Cytokines were at the center of interest and alterations in individuals with MS were observed (5–10). The chemokine interferon-γ (IFN-γ)–induced protein 10 kD [IP-10, also termed C-X-C motif chemokine ligand 10 (CXCL10)] was speculated to be a marker for inflammatory processes playing a central role in MS pathophysiology (11). IFN-γ holds a central role in the process of demyelination as it can promote the migration of leukocytes through the blood-brain barrier and stimulates astrocytes to secrete IP-10, as well as monokine induced by gamma-interferon (MIG, also termed CXCL9), C-X-C motif chemokine ligand 11 (CXCL11), and CC-chemokine ligand 2 (CCL2). These chemokines attract monocytes and promote their differentiation and subsequent production of reactive oxygen species (ROS) (7). Furthermore, MIG, IP-10, and macrophage inflammatory protein–1α (MIP-1α) are ligands of the chemokine receptor CXCR3 promoting trafficking of activated T-helper 1 (Th1) cells into the central nervous system. CXCR3 was elevated in MS patients, and MIP-1α and IP-10 were elevated within the lesions (12, 13). The results found in literature differ greatly between different studies and methodologies applied. So far, no single cytokine could be reproducibly identified as valuable biomarker for measuring the inflammatory processes in MS (9).

At the cellular level, similar heterogeneity of results was reported with respect to T-cell populations (14). There are multiple theories how certain cell types (for example, Th17 and Th1) are involved in the development of MS, but exact mechanisms are not yet understood. Nevertheless, dysregulated T cells were observed in MS patients (14, 15). Recent data further revealed that the ratio between regulatory and effector T cells distinguishes pediatric MS patients from healthy controls (16). At the genetic level, T-cell receptor (TCR) sequencing data from cerebrospinal fluid (CSF) of MS patients are still infrequent (17). To date, the primary target of the causative presumably autoreactive T cells and their receptors remain elusive.

In the present study, we investigated correlations between differentially expressed cytokines and the TCR repertoire in CSF and blood of 24 adult MS patients in comparison to nine adult control patients suffering from idiopathic intracranial hypertension (IIS). We further identified potentially relevant sequences (PRSs) associated with MS through clustering compared to a dataset of 88 individuals tagged as healthy in the immuneACCESS database (https://clients.adaptivebiotech.com/immuneaccess). Integrating cytokine and TCR data, we were aiming to display the complex nature of immune response deviation in MS beyond individually variable cytokine profiles and TCR repertoires. Our results indicated that also less significant individual cytokine deviations can contribute to a general Th1-biased type I immune response, which correlates with clonal expansion of T cells directed against EBV, CMV, and other infectious targets.

## 2 Materials and methods

### 2.1 Sample types

This study includes CSF (the cell pellet and the supernatant) and peripheral blood (serum for cytokine analysis and Ethylenediaminetetraacetic acid (EDTA)-anticoagulated whole-blood samples for TCR sequencing) referred as blood from 24 individual untreated MS patients, as well as nine patients with idiopathic IIS serving as non-inflammatory controls. All samples were frozen and stored in a biobank that follows sample handling “*in vitro* diagnostics” guidelines from the Comité Européen de Normalisation. For four subjects EDTA blood was not available. Patient data were retrieved from clinical charts. Every individual signed informed consent for biological material to be stored in the biobank and used in clinical studies. This study was conducted according to the declaration of Helsinki and approved by the ethical committee of the Austrian Red Cross (application number 20210506).

### 2.2 Cytokine analysis

Blood and CSF samples from MS and control patients were analyzed with a Bio-Plex Pro Human Cytokine Assay (Bio-Rad, Hercules, USA) to simultaneously quantify 48 different cytokines, chemokines and growth factors, including: cutaneous T-cell–attracting chemokine (CTACK), eosinophil chemotactic protein (eotaxin), fibroblast growth factor (FGF) basic, granulocyte colony-stimulating factor (G-CSF), granulocyte-macrophage colony-stimulating factor (GM-CSF), growth-regulated oncogene–α (GRO-α), hepatocyte growth factor (HGF), IFN-α2, IFN-γ, interleukin-1α (IL-1α), IL-1β, IL-1 receptor antagonist (IL-1RA), IL-2, IL-1Rα, IL-3, IL-4, IL-5, IL-6, IL-7, IL-8, IL-9, IL-10, IL-12 (p70), IL-12 (p40), IL-13, IL-15, IL-16, IL-17A, IL-18, IP-10, leukemia inhibitory factor (LIF), monocyte chemotactic protein–1 (MCP-1), MCP-3, macrophage colony-stimulating factor (M-CSF), macrophage migration inhibitory factor (MIF), MIG, MIP-1α, MIP-1β, β-nerve growth factor (β-NGF), platelet-derived growth factor (PDGF)–BB, RANTES (regulated on activation, normal T-cell expressed and secreted), stem cell factor (SCF), stem cell growth factor–β (SCGF-β), stromal cell–derived factor–1α (SDF-1α), tumor necrosis factor–α (TNF-α), TNF-β, tumor necrosis factor–related apoptosis inducing ligand (TRAIL), and vascular endothelial growth factor (VEGF). Blood samples were diluted 1:4 with sample diluent HB, and CSF samples (supplemented with 0.5% human serum albumin; Kedrion Biopharma, Barga, Italy) were measured undiluted in singlets. The multiplex magnetic bead–based assay was performed according to manufacturer’s instructions. Per analyte, 50 beads were acquired, and the fluorescence intensities were measured using the Bio-Plex 200 System and the Bio-Plex Manager software version 5.0 (Bio-Rad). All samples included in this study were tested simultaneously at the same run to achieve comparability. For each cytokine, expression values that were below the detection threshold (as specified in the manufacturer’s manual) were classified as “not detected” and further categorized by cytokine types as chemokines, growth factors, pro-inflammatory cytokines, anti-inflammatory cytokines, and other cytokines. Statistical analysis to compare cytokine expressions in MS and control patients and correlation analysis between cytokines in CSF and blood were conducted using R software (R Core Team 2024, version 4.4.1) (18).

Outliers in cytokine expression levels were identified using the interquartile range (IQR) method. For each cytokine measured within the MS and control group, the first (Q1) and third (Q3) quartiles of the expression values were computed. The IQR was calculated as the difference between Q3 and Q1. Values were then classified as outliers if they lay below Q1 minus two times the IQR or above Q3 plus two times the IQR. In the control group (n = 9), one control sample was identified as outlier and therefore removed from the analysis. Expression values were subjected to log2 transformation [log2(x + 1), where x is the raw value] to stabilize variance and improve symmetry, adding a pseudocount of 1 to accommodate potential zero values. To mitigate the potential impact of extreme measurements in Pearson’s correlation analysis, the transformed data were subsequently Winsorized using the Winsorize function within the DescTools R package. Winsorization was applied at the 12.5% level for each tail [probs = c(0.125, 0.875)]. Pairwise correlations between the processed (log2-transformed, Winsorized) cytokine levels were computed using the Pearson correlation coefficient, implemented via the rcorr function in the Hmisc R package. To account for multiple comparisons, raw p-values were adjusted using the Benjamini–Hochberg procedure to control the false discovery rate (FDR). A correlation was considered statistically significant if the absolute Pearson correlation coefficient *r* was ≥ 0.6 and the corresponding FDR-adjusted p-value was < 0.05. We then represented the correlations visually using undirected weighted graphs with iGraph package. Node colors in the graph were assigned on the basis of cytokine groups to elucidate the functional clustering of cytokine interactions. The final visualization was refined to enhance interpretability, with nodes and edges adjusted for transparency and color intensity based on their connectivity and correlation strength, respectively.

### 2.3 Isolation of genomic DNA

Genomic DNA was extracted from CSF pellets and blood samples using the Maxwell^®^ 16 LEV Blood DNA Kit (Promega, Madison, USA). First, pellets were resuspended in 200 µL of 1× Phosphate buffered saline (PBS); peripheral blood samples were thawed at 4°C. All samples were then incubated in 1× lysis buffer containing 0.1× proteinase K for 20 min at 65°C and further processed according to the manufacturer’s protocol. DNA concentration was measured using the DeNovix Spectrophotometer (DeNovix, Wilmington, USA).

### 2.4 T-cell receptor sequencing

Deep resolution immunosequencing of the CDR3 regions of human TCRβ chains was performed using the immunoSEQ assay (Adaptive Biotechnologies, Seattle, USA). Genomic DNA (5,400 ng for blood and 62–4,518 ng for CSF) was amplified in a bias-controlled multiplex PCR, followed by high-throughput sequencing to target rearranged TCR genes. Sequencing was done using V3 paired-end sequencing chemistry on the MiSeq sequencer (Illumina, San Diego, USA). Data were analyzed using the Adaptive Biotechnologies ImmunoSEQ Analyzer software (V.3.0), which identifies the V, D, and J genes, filters non-productive sequences, and reports and tracks T-cell clonality. Throughout this article, a clone is referred to as unique clone if found in an individual either in CSF or blood. If a clone is found in both CSF and blood, then it is referred to as a shared clone. A clone found in more than one individual is called a public clone. A unique or shared clone can either be public or private.

### 2.5 Identifying potentially relevant CDR3 amino acid sequences in liquor from MS patients

We defined CDR3 amino acid (CDR3.aa) sequences as PRS in MS patients if the following criteria met: (1) The sequence is 1 of the top 10 sequences regarding clone count in liquor of MS patients and has a clone count of at least two, or (2) the sequence occurs in the CSF samples of at least two different MS patients.

### 2.6 Data import, exploration in R, and identification of PRS

To import the sequencing data from immunoSEQ into R, the library tool immunarch was used. Because the number of healthy controls in our dataset was rather limited, we acquired a dataset with the samples of 88 individuals tagged as healthy from immunoSEQ database (ImseqHealthy). The immunarch dataset was filtered for samples that occurred within CSF of MS patients only. Because we decided to look specifically for CDR3.aa without taking the clonotype into account, the clone count of identical CDR3.aa with different clonotype was summed up. Overall, 23 sequences fulfilling these search criteria could be identified. For the identification of CDR3.aa occurring in more than one CSF sample of the MS group, the immunarch function pubrep was used. In sum, 186 PRSs were identified.

### 2.7 Filtering for PRS

Using the identified PRS, we searched the whole dataset (including CSF and blood from MS and ImseqHealthy individuals) for the defined identical CDR3.aa sequences. To identify sequences associated with MS, we used k-means clustering. The clone count from each sample was ordered for every distinct PRS by rank. This ranking was used as input for k-means clustering to identify two clusters (k = 2), assigning each PRS to cluster 1 or cluster 2. Because clustering was done with 109 dimensions (88 clone count ranks from healthy and 21 from MS individuals), dimensionality reduction with principal component analysis (PCA) was done to visualize clusters. CDR3.aa sequences in the MS cluster were searched in McPAS database for disease association (19).

### 2.8 Data analysis and statistics

Data analyses regarding TCR were conducted using the statistical software R (version 4.3.2) (18). Differences of clones in clusters 1 and 2, respectively, within the two-sample groups (MS and healthy individuals) were analyzed using a t-test with Welch correction with p < 0.05 assumed being significant. Graph Pad Prism 10 (GraphPad Software, Inc., La Jolla, USA) was used for statistical analysis and graphs regarding cytokine profiling. In addition, force-directed correlation network graphs and cytokine heatmap were generated with Python 3.10.8. Pandas 2.0.0 was used for tables, Numpy 1.24.1 (20) for scaling, Seaborn 0.12.2 (21) for generating the heatmap, and Matplotlip 3.7.1 (22) for depiction of the heatmap. For the correlation network graphs, Pearson correlations were calculated with Pandas 2.0.0 and created using NetworkX 3.1 (23). Regarding heatmaps of cytokine profiles, a logarithmic value with a base of 2 was calculated for every data set. Data were scaled in a logarithmic manner according to mean and standard deviation. For each cytokine, the mean was normalized to 0 using the calculation [(value − mean)/standard deviation] to obtain a comparable scale.

### 2.9 Analysis of T-cell receptor sequences and their correlation with cytokine expression in MS patients

To link the relevant TCR sequence proportions to the cytokine expressions, we first identified the TCR sequences exclusively present in at least five MS patients or present in both MS and controls but with at least a 10-fold enrichment of clone proportions in MS compared to controls. Kendall’s tau was used to correlate selected TCR sequences with cytokine levels, identifying significant relationships (Benjamini–Hochberg multiple testing correction, adjusted p < 0.05 and |correlation coefficient| > 0.5). This method is robust to non-parametric distributions, suitable for the ordinal nature of sequence data.

## 3 Results

### 3.1 Characteristics of the study cohort

Twenty-four MS patients and nine patients with idiopathic IIS serving as controls were included in the present study. An additional control dataset from 88 individuals including 8,022,194 additional sequences considered as “healthy” was utilized for TCR analysis. All MS patients were untreated, and lumbar puncture was performed at the first presentation for diagnostic purposes. Age and sex distribution was comparable between MS and controls (**Supplementary Figure S1**). The median Expanded Disability Status Scale (EDSS) (24) of MS patients at time of presentation was 1.

### 3.2 Cytokine expression pattern intrathecal vs. peripheral blood compartment

First, we measured 48 different cytokines in CSF and blood from MS and control patients. We categorized the cytokines into five functional groups (25–28): chemokines, growth factors, pro-inflammatory cytokines, anti-inflammatory cytokines, and other cytokines based on their role in the immune response and in other physiological processes (**Table 1**).

**Table 1:** Cytokines grouped according to their respective functional categories.

Functional category	Cytokines
Chemokines	CTACK, eotaxin, GRO-α, IL-8, IP-10, MCP-1, MCP-3, MIG, MIP-1α, MIP-1β, RANTES, and SDF-1α
Growth factors	FGF2, G-CSF, GM-CSF, HGF, SCF, SCGF-β, VEGF, β-NGF, and PDGF-BB
Pro-inflammatory	IFN-γ, IL-1α, IL-1β, IL-6, IL-12, IL-12-1, IL-17, IL-18, TNF-α, and TNF-β
Anti-inflammatory	IL-1RA, IL-4, IL-10, and IL-13
Other	IFN-α2, IL-2, IL-2Rα, IL-3, IL-5, IL-7, IL-9, IL-15, IL-16, LIF, M-CSF, MIF, and TRAIL

**Figure 1:**
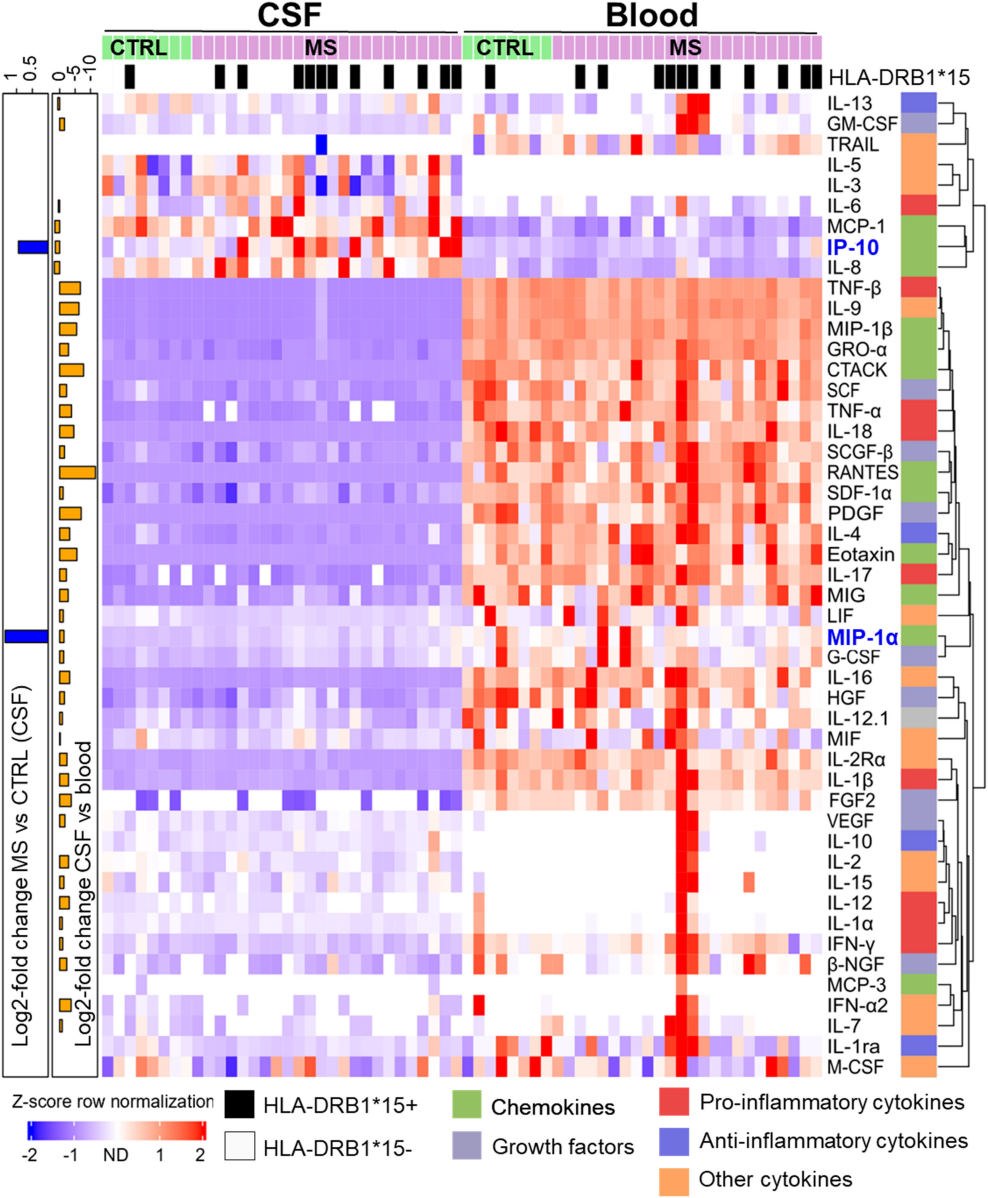
Differential cytokine expression pattern in CSF and blood of MS and control (CTRL) patients. Samples were sorted column-wise as indicated. HLA-DRB1*15:01 positive (+) patients are marked at the top with a black square. Cytokine expression levels were normalized row-wise using Z-score and clustered by Euclidian distance metrics. Violet/blue Z-score indicates lower and red indicates higher expression. Values shown in white were not detected. Cytokines were classified according to their predominant biological function as color-coded on the right (see **Table 1**). The dendrogram at the right indicates the degree of similarity in expression patterns by the length of the branches. Cytokines with more similar expression levels cluster together with shorter branch lengths. At the left y-axis, significant log2-fold differences between MS vs. CTRL in CSF (blue bars) and between CSF and blood sample types (orange bars), respectively, are shown. The scale on top left indicates the amplitude and the direction of the log2-fold change (Mann–Whitney U-test, adjusted p < 0.05).

Using stringent statistics, we found CSF of two cytokines in CSF are significantly higher in MS patients compared to that in controls (MIP-1α, 2.57-fold, and IP-10, 1.91-fold, respectively) (**Supplementary Figure S2**). We used Z-scoring to normalize data enabling a systematic comparison of the highly variable levels of different cytokines in CSF and blood of MS and controls patients, respectively. Individual cytokines were clustered using Euclidean distance, to group cytokines based on their expression, in rows. Two major clusters were detected with the top one showing a scattered higher cytokine expression in CSF compared to blood (**Figure 1**).

The second cluster is composed of cytokines with higher expression in blood compared to CSF in most patients. The lower proportion of the second cluster also contained cytokines, which were detected in just a few patients with higher expression in blood compared to CSF. Overall, most cytokines showed a stronger signal in blood compared to CSF. TRAIL was predominantly found in blood whereas IL-3 and IL-5 just in CSF. No additional significant difference was found between MS and control patients in blood. We confirmed a significantly increased prevalence of DRB1*15:01 in MS patients (**Figure 1**).

### 3.3 Correlation of cytokines

**Figure 2:**
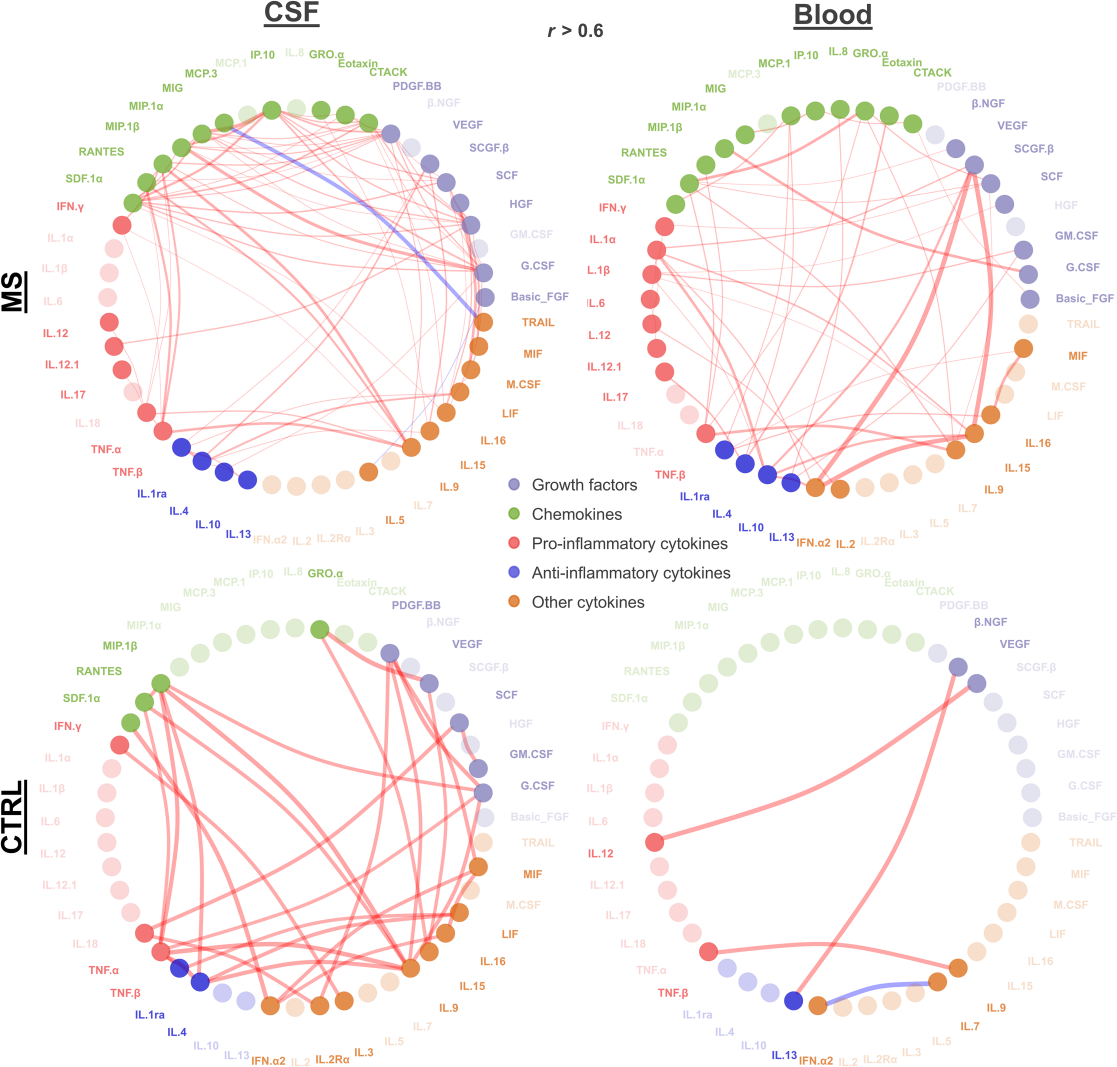
Cytokine correlation. Significant correlations (absolute r > 0.6 and adjusted p < 0.05) between cytokines in MS and control patients for CSF and blood are depicted. Each node represents a cytokine connected by lines representing the significant correlation (red, positive correlation; blue, negative correlation). Line width reflects the correlation strength. Node color indicates growth factors, chemokines, and pro- vs. anti-inflammatory and other cytokines as shown. Nodes with higher transparency levels did not have any significant correlation.

To question connectivity of cytokine profiles, we performed Pearson’s correlation of log2 cytokine levels. In MS patient’s CSF, 77 significant positive and two negative correlations (*r* > 0.6, adjusted p < 0.05) were found. IP-10–MIG, G-CSF–MIP-10, and IL-9–MIP-10 demonstrated strongest positive correlation (*r* > 0.87 for all three pairs), whereas MCP3–TRAIL was strongly negatively correlated (*r* = −0.95). G-CSF, PDGF-BB, and HGF correlated with 11, 10, and 8 other cytokines, respectively. In MS patient’s blood, 45 significant positive correlations (*r* > 0.6, adjusted p < 0.05) were identified. VEGF–IFN-α2 exhibited the strongest correlation (*r* = 0.99) followed by VEGF–IL-15 (*r* = 0.99) and IFN-α2–IL-15 (*r* = 0.99). IL-1α, IL-10, and IL-15 were found to correlate with six, five, and five other cytokines, respectively (**Figure 2**).

Overall, we found distinct correlations within the compartments CSF and blood with certain cytokines being correlated in just one compartment, such as TNF-α, TRAIL, PDGF-BB, and IL-5 in CSF, or IFN-α2, IL-2, GM-CSF, and β-NGF in blood. Some cytokines did not show any correlation in both blood and CSF (IL-1α, IFN-α2, IL-2R α, IL-7, IL-3, IL-7, and β-NGF). We observed more pro-inflammatory showing significant correlation in CSF compared to that in blood. For the control group, in CSF, we found 31 significant positive correlations (r > 0.6, adjusted p < 0.05). The most robust correlations were VEGF–GRO-α (*r* = 0.99), IL-9–TNF-β (*r* = 0.992), and TNF-β–IL-4 (*r* = 0.990). IL-9, TNF-α, and IL-1RA demonstrated the highest number of correlations, each correlating with six other cytokines. In control blood, significant positive correlations (r > 0.6, p < 0.05) were identified, along with one negative correlation. The strongest positive correlation was observed between VEGF–IL-12 (*r* = 1), followed by IL-1RA–MCP-1 (*r* = 0.950) and TNF-β and IL-9 (*r* = 0.937) (**Figure 2**).

**Figure 3:**
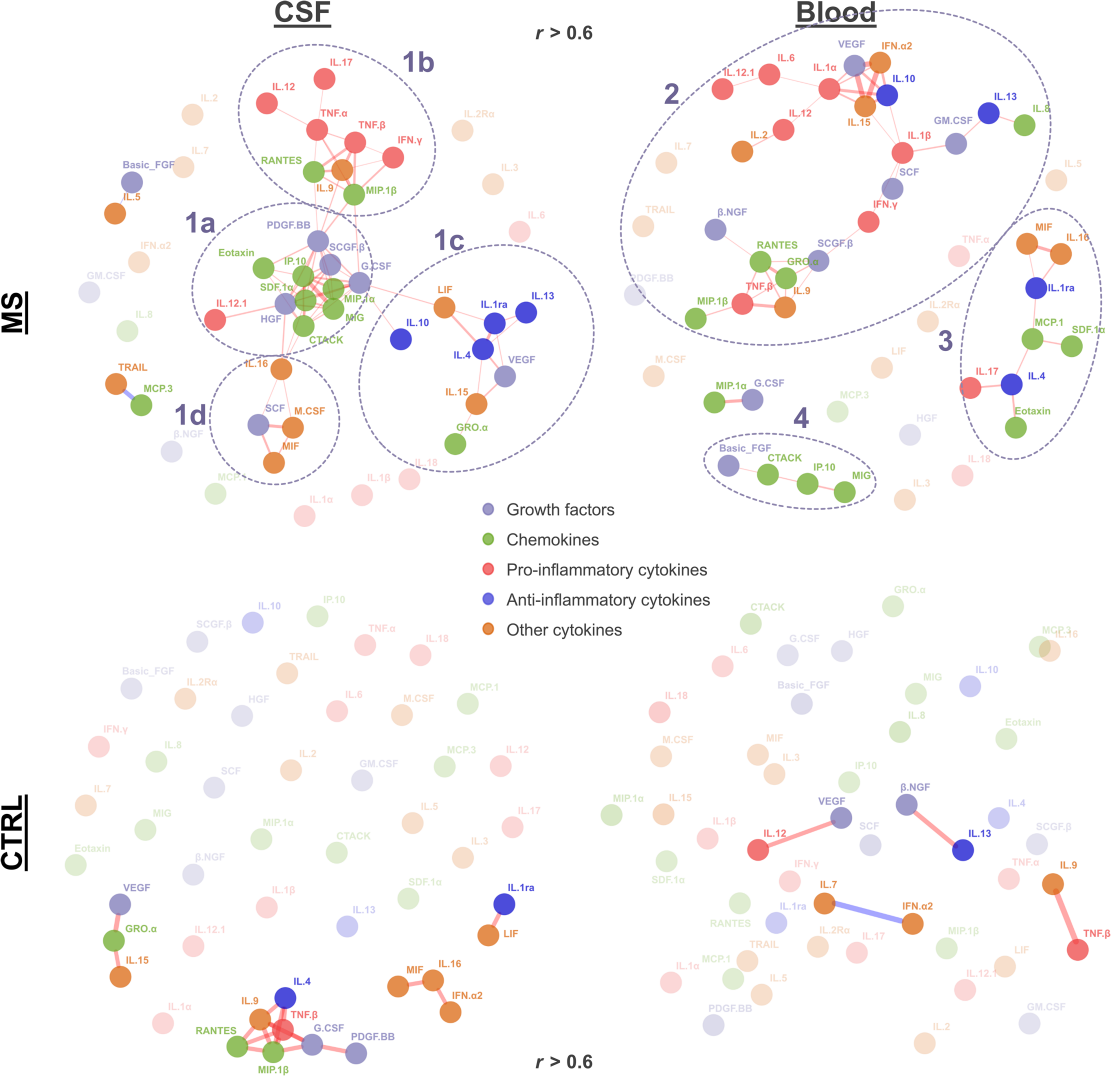
Cytokine correlation network. The force-directed network (Fruchterman–Reingold algorithm) displays significant correlations (r > 0.6, adjusted p < 0.05) between cytokines. Each node represents a cytokine, and the lines represent significant correlation (red, positive correlation; blue, negative correlation). Node color indicates growth factors, chemokines, and pro- vs. anti-inflammatory and other cytokines as indicated. Line width reflects the correlation strength. Nodes with higher transparency levels and transparent text do not have any significant correlation. Clusters identified in MS patients are circled with dashed lines and numbered (1a–1d, 2, 3, and 4).

To visualize individual clusters of cytokines correlating with each other, we used a force-directed algorithm (**Figure 3**). In MS patients’ CSF, we identified one major cluster composed of four sub-clusters [cluster 1a (mostly chemokines and growth factors): G-CSF, MIP-1α, IP-10, SDF-1α, HGF, IL-12, SCGF-β, CTACK, MIG, PDGF-BB, M-CSF, and SCF; cluster 1b (mostly pro-inflammatory): MIP-1β, TNF-α, IFN-γ, TNF-β, RANTES, IL-9, IL-17, and IL-12; cluster 1c (high proportion of anti-inflammatory cytokines): IL-1RA, IL-10, IL-13, IL-4, LIF, IL-15, VEGF, and GRO-α; and cluster 1d (mostly other cytokines): MIF, M-CSF, SCF, and IL-16].

In MS patients’ blood, we identified another two clusters. Cluster 2 contained a mix of pro-inflammatory (IFN-γ, IL-1β, IL-1α, IL-12, TNF-β, IL-6, and IL-12.1), anti-inflammatory (IL-10 and IL-13), growth factors (SCGF-β, SCF, GM-CSF, VEGF, and β-NGF), chemokines (RANTES, GRO-α, IL-8, and MIP-1β), and other cytokines (IL-2, IL-9, IL-15, and IFN-α2). Cluster 3 also contained a mix of chemokines (Eotaxin, MCP-1, and SDF-1α), anti-inflammatory cytokines (IL-4 and IL-1RA), pro-inflammatory cytokines (IL-17), and the other cytokines MIF and IL-16. The smaller cluster 4 comprised FGF2, CTACK, IP-10, and MIG. In control patients, we observed smaller networks in CSF (the biggest cluster containing TNF-β, IL-4, RANTES, MIP-1β, IL-4, IL-9, G-CSF, and PDGF-BB), whereas no clusters were found in blood (**Figure 3**).

### 3.4 TCR repertoire

**Figure 4:**
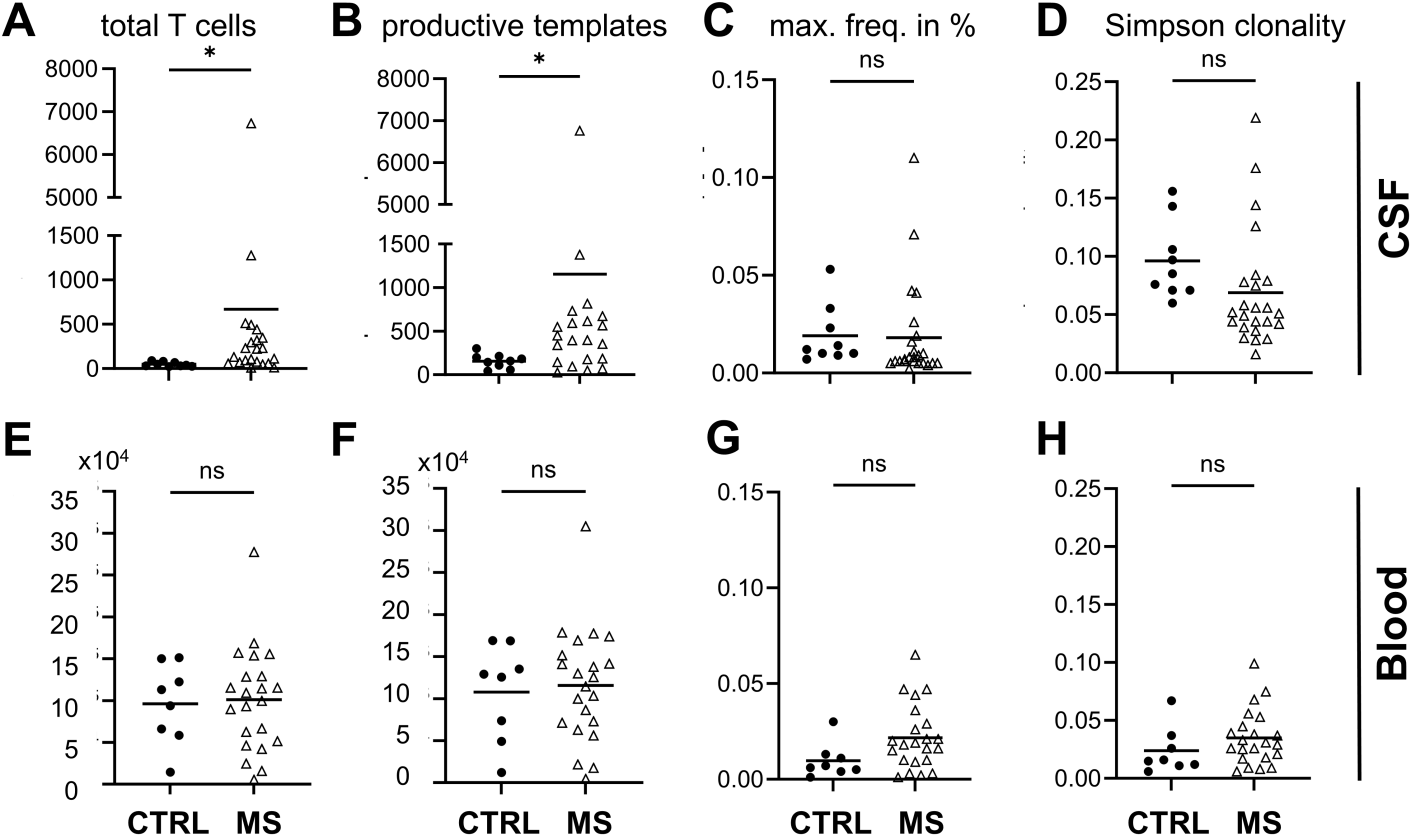
T-cell analysis. CSF data top, blood data lower row as indicated. **(A, E)** T-cell numbers, **(B, F)** productive templates and **(C, G)** maximum frequency (max. freq.) given in percent, and **(D, H)** Simpson clonality index obtained by T-cell receptor sequencing of CSF and blood from MS and control patients. Welch’s t-test *p < 0.05; ns, not significant; MS n = 22.

The total number of T cells (**Figure 4A**) and the number of productive templates (**Figure 4B**) were significantly higher in CSF of MS patients. The maximum frequency of clones was comparable in both groups in CSF (**Figure 4C**). Simpson clonality index for diversity was not significantly different between MS and control patients (**Figure 4D**). In blood, we observed no significant differences regarding total number of T cells, productive templates and rearrangements, maximum frequency, and Simpson clonality index between MS patients and controls (**Figures 4E–H**).

**Figure 5:**
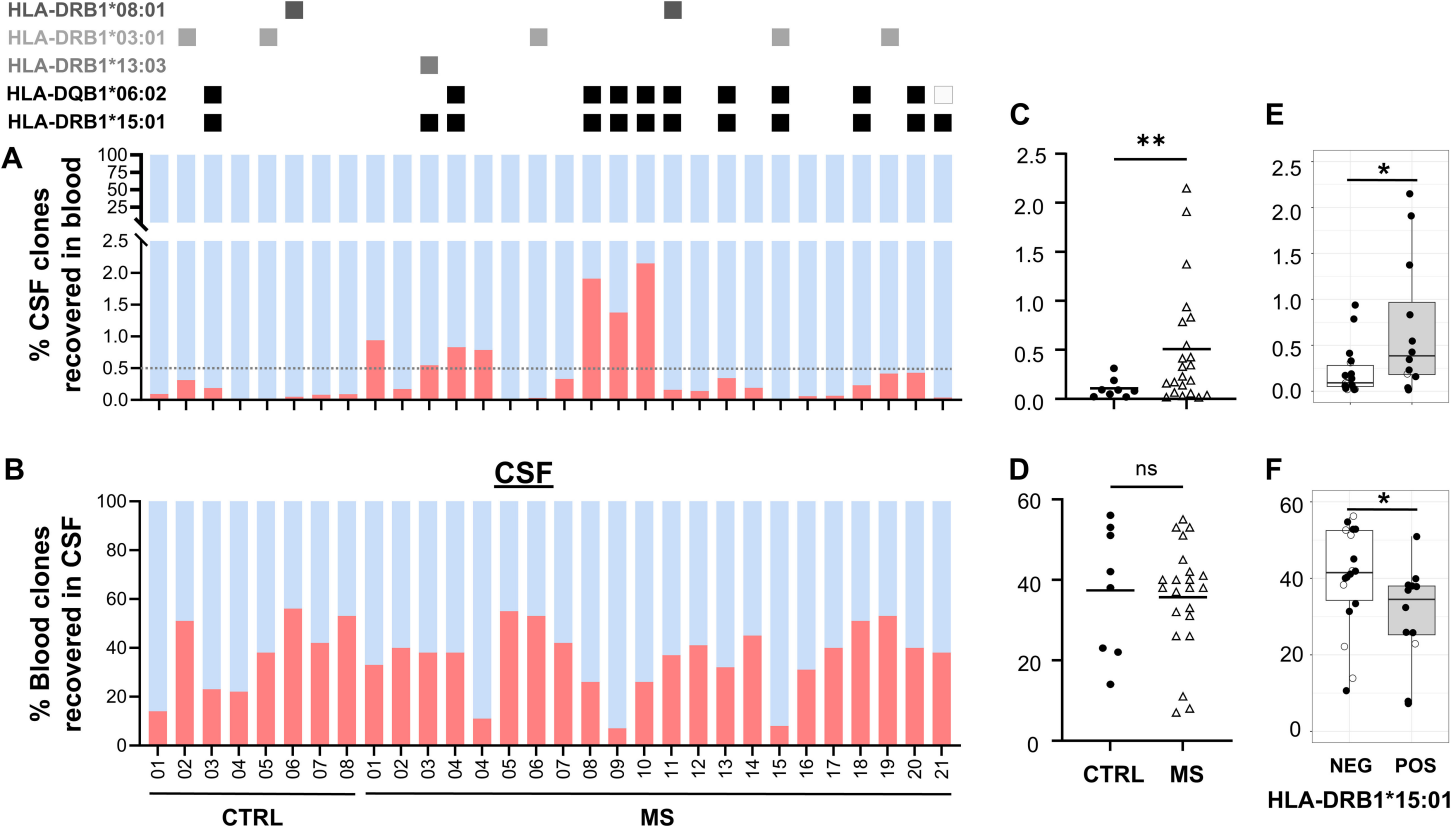
Shared and unique T-cell clones. Percentages of T-cell clones that are shared (red) or unique (blue) between both sources **(A)** in blood and **(B)** in CSF. MS patient and CTRL patient ID at the x-axis. HLA-DRB1*15:01–positive patients are marked at the top with a black square. Three patients whose blood was not available for TCR sequencing were excluded. Percentages of shared T-cell clones in **(C)** blood and **(D)** CSF. Percentages of shared T-cell clones in HLA-DRB1*15:01 positive (pos) vs. negative (neg) patients in **(E)** blood and **(F)** CSF. Unpaired T-test and Welch’s F-test revealed significant differences despite different sample size; *p = 0.0266, **p<0.005; ns, not significant.

The recovery of shared clones in blood, which were initially identified in CSF, was significantly higher in MS patients (MS patients’ mean = 0.5% versus control patients’ mean = 0.1%, p = 0.0064) corresponding to a cerebral T-cell–mediated pathology with clonal expansion (**Figures 5A, C**). The most frequent HLA class II allele in the MS patient group was HLA-DRB1*15:01 (11 out of the 22 MS patients), which was found in just one control patient (Fisher’s exact test, p = 0.0994, odds ratio = 6.61). The second most common allele was HLA-DQB1*06:02 found in 9 out of the 22 MS patients and in one control patient, in linkage disequilibrium with HLA-DRB1*15:01 (29). Other known MS predisposing HLA alleles were also detected in MS and at lower proportion in control patients. The number of T cells and the overlap in patients showed a high variability (range, 0.015–2.149). In seven patients, the recovery of CSF-detected clones in blood was >0.5%, but no correlation was found with disease severity measured with EDSS at presentation, number of cells in CSF, age, or gender, in these patients. In MS patients, mean = 35.7% of clones identified in blood were retrieved in CSF, compared to mean = 37.4% in control patients, indicating that the number of shared clones in blood alone is not sufficient to discriminate MS from control patients (**Figures 5B, D**). We found significantly more shared CSF-detected clones recovered in blood and significantly less shared blood-detected clones recovered in CSF of HLA-DRB1^+^ MS patients (**Figures 5E, F**).

**Figure 6:**
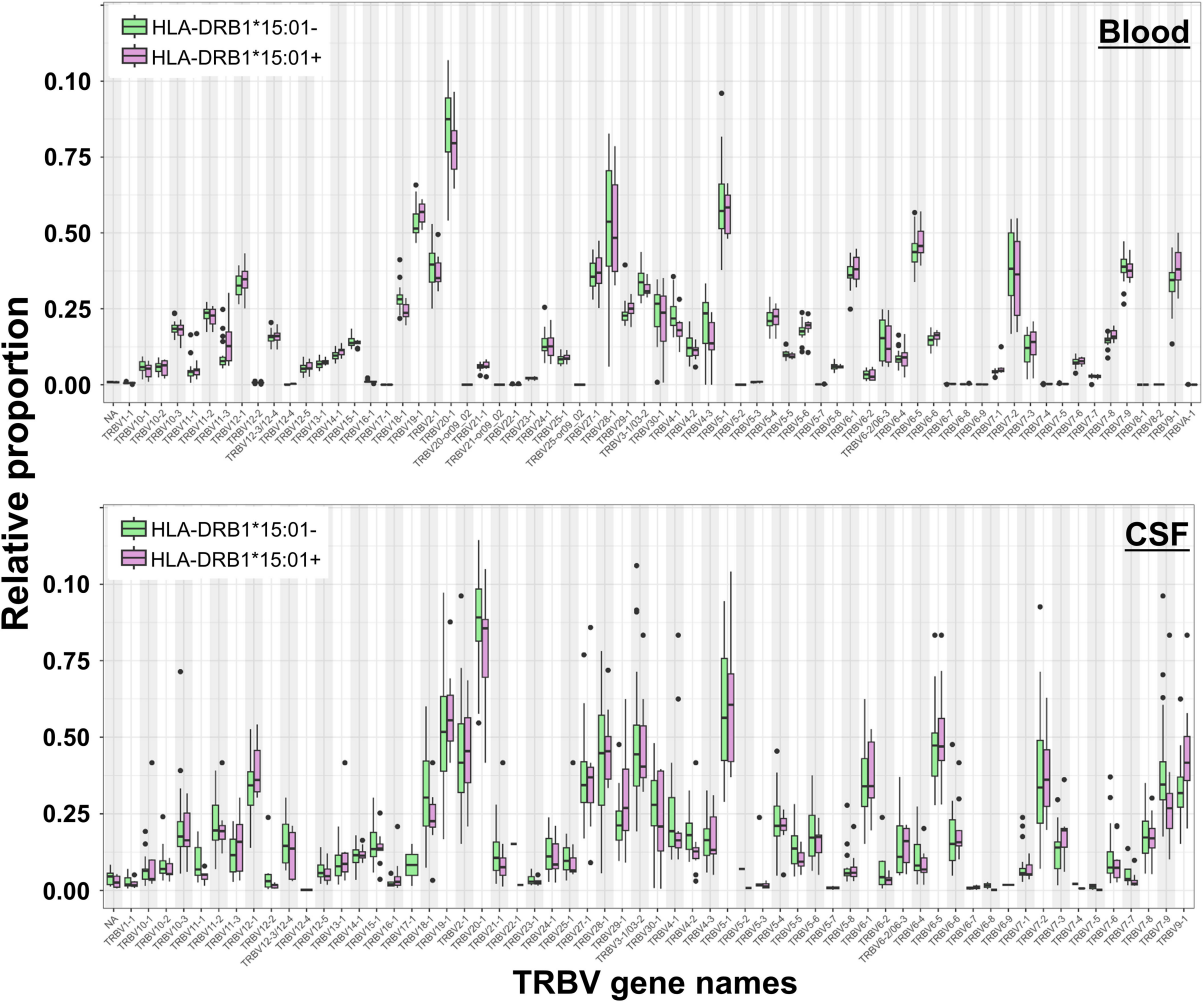
T-cell receptor beta variable (TRBV) gene usage in blood and CSF of HLA-DRB1*15:01–positive and HLA-DRB1*15:01–negative patients. Box plots showing the relative proportions of TRBV gene usage in blood (top) and CSF (bottom) samples from HLA-DRB1*15:01–positive (purple boxes) compared to HLA-DRB1*15:01–negative patients (green boxes). No significant differences were found (Mann–Whitney U-test, Benjamini-Hochberg (BH) adjusted p < 0.05).

We performed an additional gene usage analysis to assess if clonotypic TCR beta variable (TRBV) genes were over- or under-represented in MS compared to that in control patients. This approach involved quantifying the frequency of each TRBV gene segment in the T-cell population, allowing us to detect significant differences in TRBV expression between CSF and blood of MS patients. In CSF of MS patients, we did not find significant changes in TRBV usage between MS and control patients in both blood and CSF (**Supplementary Figure S3**). We also showed no significant differences between HLA-DRB1*15:01^+^ and HLA-DRB1*15:01^−^ patients, respectively (**Figure 6**). TRBV gene representation in the different TCR-cytokine correlation groups revealed different distribution patterns across the different cytokine groups. We identified the most variable TRBV: TRBV28-1, TRBV6-5, TRBV5-6, TRBV3-1, TRBV27-1, TRBV19-1, TRBV7-9, TRBV12-1, TRBV20-1, and TRBV7-2. TRBV28–1 and TRBV5–6 showed the lowest proportion in the anti-inflammatory group, whereas TRBV6–5 showed the highest compared to that in the other groups (**Supplementary Figure S6**).

### 3.5 Correlation between blood TCR repertoire and cytokine profile in MS

Finding significantly more shared clones detected in CSF to be recovered in blood (**Figure 5A**) in significantly more patients bearing HLA-DRB1*15:01 (**Figure 5E**) prompted us to correlate the blood TCR repertoire with blood cytokine profile. After pre-filtering TCR data to select sequences present in at least two samples, 166,569 public CDR3.aa sequences were thus identified. We next selected MS-”predominant” CDR3.aa sequences that were either found in at least five MS patients but not in controls or that were present in both MS patients and controls but with at least a 10-fold enrichment in MS patients. This yielded 2,644 MS-”predominant” TCR sequences in MS patient’s blood. Correlation analysis between the MS-”predominant” sequences and the 48 cytokines in MS patient’s blood identified 665 significant associations involving 492 distinct sequences and 34 cytokines (absolute Kendall correlation > 0.5, p < 0.05). Thereof, 391 showed positive and 274 showed negative correlations.

Among the significant correlations, 628 were with 465 distinct CDR3.aa sequences found only in MS patients, whereas 37 correlations were with 27 distinct CDR3.aa sequences found both in MS and control patients. The cytokines showing most enriched significant correlation with CDR3.aa sequences in MS, compared to those in control patients, were SCF with CASSHQETQYF (7.1 log2-fold change), SDF-1α with CASSYSTGGYGYTF (6.1 log2-fold change), IP-10 with CASSYSTNSYEQYF (5.7 log2-fold change), TRAIL with CASSYSGDQPQHF (5.3 log2-fold change), and eotaxin with CASSELAGGTDTQYF (4.9 log2-fold change).

**Figure 7:**
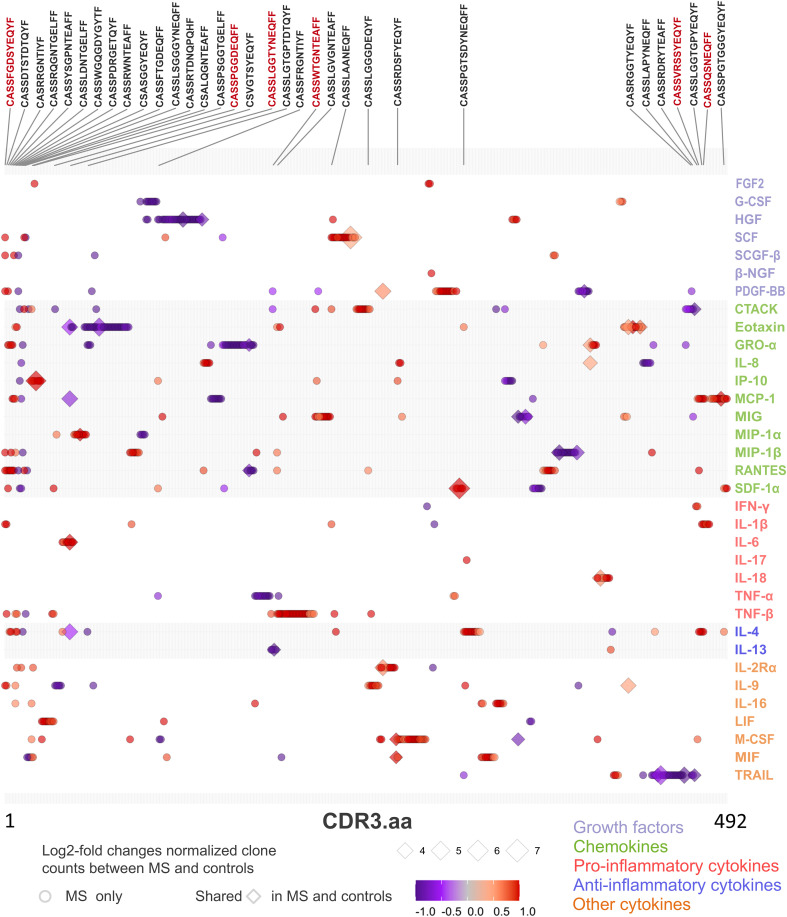
TCR CDR3 amino acid (aa) sequence clone count and cytokine paired correlations in MS patient’s blood. Dot chart illustrating correlation between TCR sequences (CDR3.aa log2-fold changes) and cytokine expression level in blood of MS patients. We selected 2,644 MS-”predominant” sequences found in at least five MS patients but not in controls (circles) or in both MS and control patients with at least ten-fold enrichment for the normalized clone count analysis in MS (diamonds). Each point represents a unique significant TCR sequence-cytokine pair (adjusted p < 0.05, Kendall rank correlation > 0.5). Symbol color indicates strength and direction of correlation as indicated in the legend and the size of each point reflects the log2-fold enrichment of normalized clone counts in MS patients compared to controls. Sequences that have significant correlation with more than two cytokines are shown above the chart. Red text color indicates the six sequences that have a 100% match in the McPAS database (**Table 3**).

We found 52 predicted CDR3.aa sequences correlating with eotaxin (18 positive and 34 negative), 38 with GRO-α (10 positive and 28 negative), 37 with HGF (5 positives and 32 negatives), 35 with TNF-β 35 (34 positive and 1 negative), and 32 with MCP-1 (21 positives and 11 negatives), respectively. IL-17 and β-NGF correlated with only one CDR3.aa sequence each (**Figure 7**). At the functional level, growth factors and chemokines showed a balanced proportion between positive and negative correlations (53/58 and 152/147 positives/negatives, respectively), whereas pro-inflammatory and anti-inflammatory showed a higher proportion of positive (66/16) compared to negative correlations (25/8) (**Table 2**). Individual CDR3.aa sequences correlated with multiple cytokines (e.g., CASSFGDSYEQYF and CASSPDRGETQYF with seven). We identified 33 CDR3.aa sequences correlating with at least three cytokines; among these, six showed 100% matches in the McPAS database. Overall, 36 out of the 492 significantly correlated sequences had 100% matches in McPAS mainly addressing influenza M1, yellow fever, and *Mycobacterium tuberculosis* targets (**Table 3**). Less than 30 correlations total were identified in control patients due to the low number of controls and the fact that not all TCR sequences were found in all patients (**Supplementary Figure S4**). We also found a significantly higher number of correlations between TCR sequences and chemokines (median = 24) than that of between TCR sequences and anti-inflammatory cytokines (median = 9; **Supplementary Figure S5**).

**Table 2:** Number of positive and negative correlations between TCR sequences and the functional cytokine groups.

Category	Positive correlations	Negative correlations
Growth factors	53	58
Chemokines	152	147
**Pro-inflammatory cytokines**	**66**	**16**
**Anti-inflammatory cytokines**	**25**	**8**
Other cytokines	95	45
Total	391	274

**Table 3:** Public CDR3.aa sequences found correlating with cytokines in blood with McPAS matches.

CDR3.aa sequence	MS patients’ blood (percentage)	MS patients’ CSF (percentage)	Association with
CASSFGDSYEQYF	20.83	0	Yellow fever virus
CASSGGYGYTF	25	0	*M. tuberculosis*
CASSPGGDEQFF	25	4.17	*M. tuberculosis*
CASSLGGTYNEQFF	41.67	0	*M. tuberculosis*
CASSSGQKNTEAFF	20.83	8.33	Cytomegalovirus
CASSPGTTTNEKLFF	20.83	0	Neoantigen
CASSIDRGYGYTF	20.83	0	HTLV-1
CASSVGGGSNQPQHF	25	0	Neoantigen
CASSIRSNQPQHF	20.83	0	Influenza
CASSLSNYEQYF	25	0	Influenza
CASSFGGSYNSPLHF	20.83	0	Influenza
CASSRQSNQPQHF	25	0	*M. tuberculosis*
CSARAGGNTEAFF	25	0	*M. tuberculosis*
CASSWTGNTEAFF	41.67	0	Neoantigen
CASSIGTDTQYF	20.83	0	Alzheimer’s disease
CASSYGSYNEQFF	29.17	0	Tumor-associated antigen
CASSRTGGIYEQYF	20.83	0	Toxic epidermal necrolysis
CASSLLADYNEQFF	20.83	0	Influenza
CASSARGGTDTQYF	20.83	0	Influenza
CASSRGQGEQYF	20.83	0	Epstein–Barr virus
CASSPSSTDTQYF	41.67	0	*M. tuberculosis*
CASSSTAYEQYF	20.83	0	Influenza
CASSPGESYEQYF	20.83	0	Cytomegalovirus
CASSQDLAGGPDTQYF	25	0	*M. tuberculosis*
CASSWGGEQYF	20.83	0	Influenza
CASSETSGGADTQYF	41.67	0	*M. tuberculosis*
CASSGTSGGTDTQYF	45.83	4.17	*M. tuberculosis*
CASSLTDEQYF	25	0	Influenza
CASSLEGVSTDTQYF	20.83	0	*M. tuberculosis*
CAWSRDSGSGNTIYF	25	0	*M. tuberculosis*
CASSSTGGGETQYF	25	0	Rheumatoid arthritis
CASSERETQYF	37.5	8.33	*M. tuberculosis*
CSARGQETQYF	20.83	0	*M. tuberculosis*
CASSVRSSYEQYF	20.83	0	Influenza
CASSVRSSYEQYF	20.83	0	Cytomegalovirus
CASSQSNEQFF	20.83	4.17	*M. tuberculosis*
CASSSQGAYGYTF	25	0	Yellow fever virus

The sequences listed are found to be associated with certain viruses or diseases without target identification.

The k-means clustering of PRS revealed two clusters (**Supplementary Figure S7**). Sequences in the MS cluster (138 sequences, **Supplementary Table S1**) were significantly more often found in MS patients (mean 22.3%) than in healthy controls (mean = 4.1%; t-test, p = 0.024). The pathology-associated TCR database McPAS (19) revealed 13 sequences with known association assigned to the MS cluster. Three thereof were assigned to EBV, two targeting the BMLF1 (30), and one targeting EBNA-3A (31) (**Table 4**). When comparing the mean frequencies of sequences assigned to the general cluster (blue, not restricted to MS; **Supplementary Figure S7**), no significant differences were found (MS, mean = 4.8%; general, mean = 4.5%; t-test, p = 0.72). Among the 138 sequences identified in the MS cluster, four were also found correlating with cytokines (CASSQQGNYGYTF positively correlating with TNF-β, CASSLAGRGQETQYF negatively correlating with HGF, CASRRNTGELFF negatively correlating with TRAIL, and CASSQGLADYNEQFF positively correlating with G-CSF).

**Table 4:** MS cluster PRS CDR3.aa sequences and their maximum frequency in CSF.

CDR3.aa sequence	MS patients’ CSF (percentage)	Max. frequency	Target/association
CASSDSSTDTQYF	12 (50.0%)	23	EBV BMLF-1
CASSLDTDTQYF	13 (54.2%)	5	EBV BMLF-1
CASSQDRLTGGYTF	3 (12.5%)	1093	EBV EBNA-3A
CASSLEGETQYF	11 (45.8%)	10	Cytomegalovirus/Influenza
CASSLGVGQPQHF	9 (37.5%)	54	Melan-A/MART-1
CASSLRDSSYEQYF	5 (20.8%)	3	Diabetes mellitus (m)
CASSSGQKNTEAFF	5 (20.8%)	9	Cytomegalovirus
CASSSGYGYTF	16 (66.6%)	7	Influenza
CASSSNEKLFF	11 (45.8%)	2	*M. tuberculosis*
CASSYGGEQYF	7 (29.2%)	4	Influenza
CASSYSSGGADTQYF	8 (33.3%)	44	*M. tuberculosis*
CASSERETQYF	9 (37.5%)	5	*M. tuberculosis*
CASSPGYSNQPQHF	9 (37.5%)	2	*M. tuberculosis*

## 4 Discussion

In this study, we analyzed 48 cytokines and the TCR repertoire in CSF and blood of MS and control patients, respectively. Despite expected inter-patient variability in CSF T-cell count and cytokine expression, our individual primary datasets on cytokine profiles and TCR repertoire are in general agreement with published results (5–10, 12, 17). We found MIG, MIP1a, and IP-10 at significantly higher, and LIF and IL13 at significantly lower levels in CSF of MS compared to that of control patients. We confirmed the HLA-DRB1*15:01 prevalence and determined a significant TRBV gene preference in MS patients in our study population.

Currently available data on cytokine expression in MS differ widely, and no single cytokine was proven as suitable disease activity biomarker so far (8, 9, 32, 33). Therefore, our focus in the present study was to gain an overview of cytokine expression and TCR profiles in MS patients comparing CSF and blood, respectively. We found a significantly higher recovery rate of shared TCR sequences in blood initially identified in CSF of MS patients in agreement with a peripheral activation of central nervous system (CNS)–reactive T cells (1). We identified 2,644 shared TCR sequences correlating with predominantly pro-inflammatory cytokines, e.g., 34 CDR3.aa sequences positively correlating with TNF-β. Additional correlation with chemokines might indicate the more widespread inflammation related to innate immune response components (2).

### 4.1 Limitations

The small sample size of MS and control patients is a limitation of this study restricting statistical power particularly of high-dimensional analysis of cytokine-TCR correlation. We still found significant differences between the two groups. Available control data include TCR sequences but lack cytokine data in the same patients; therefore, it was not possible to include an independent validation set to this study. TCR clones could not be linked to T-cell types and their functionality or, respectively, cytokine expression profile as we did not perform single-cell sequencing. Our bio-banked samples were cryopreserved and, therefore, functional assays to prove specificity could not be performed.

Further, Idiopathic intracranial hypertension (IIH) patients were used as controls in this study despite evidence that this disease is not immunologically inert; IL-2 and IL-17 were found in CSF of IIH patients at higher levels (34). In our study, IL-2 and IL-17 were found elevated in serum of MS and control patients in comparison to CSF. Finally, detailed vaccination records were not available for our study cohort; also McPAS does not give further information about the sequences except being in association with certain diseases. We did not focus on viral specificity in this study.

### 4.2 Cytokine profiles

We identified elevated levels of IP-10 and MIP-1α in CSF of MS patients. As IP-10 and MIP-1α can promote migration of Th1 cells across the blood brain barrier through activation of CXCR3, our results may reflect a typical pattern in MS pathogenesis in line with other studies (5, 12, 13, 32, 35, 36). Overall, we found elevated levels of IL-13, IL-6, MCP-1, IP-10, and IL-8 in CSF in comparison to blood of MS patients and controls. Higher levels of eotaxin and IL-2RA could not be reproduced in our limited dataset (10). In previous studies, significantly different expression patterns were observed for TNF-α and IL-17 in MS patients (9, 37, 38). Such a difference was not observed in our sample set. This may be explained by different analysis methods applied, known study heterogeneity (9), pre-analytical sample handling, as well as patient cohorts. Berek et al. (10) recently also described a significantly different cytokine profile in CSF and blood of MS patients at primary diagnosis. IP-10, MCP-1, and IL-8 were found elevated among 29 total cytokines increased in CSF of their patient. Because their study did not include a control group, we may not speculate whether a cytokine network analysis of their data would result in identification of a comparably sparse network in CSF. In a meta-analysis (9) comparing cytokine levels in MS patients to those in control subjects, 13 cytokines were found to be elevated in the CSF of MS patients. Notably, MIP-1α (also known as CCL3) was found to be associated with MS in our data as well. It has to be noted that the cytokines tested just partly overlap between the studies.

### 4.3 Cytokine correlation

Comparing cytokine correlations, we found an obviously narrowed correlation network in CSF of MS patients compared to control patients. The CSF cytokine correlation network in MS showed compartmentalized cytokine clusters, potentially reflecting immunologically confined immune responses within the CNS. We found different cytokine clusters in MS patients. The dominant cluster 2 in MS blood encompassed a mix of pro- and anti-inflammatory cytokines, growth factors, and chemokines in accordance with a dysregulated immune response (1). The presence of pro-inflammatory cytokines like IFN-γ, TNF-α, and IL-17 underscores the inflammatory nature of MS (39). Growth factors like PDGF-BB and chemokines such as MCP-1 and IL-8 may be associated with ongoing tissue damage and immune cell recruitment to the CNS (40).

Correlation analysis of our data revealed clearly dysregulated cytokine profiles with substantially weaker clustering in CSF and blood samples of MS patients compared to control samples, with the latter showing more balanced cytokine profiles with strong interconnection between single factors. A cytokine imbalance in MS patients may be of particular interest and should be taken into account when analyzing cytokine levels in MS patients for diagnostic purposes or for monitoring therapy response. Furthermore, this might explain why targeting a single cytokine in therapeutic approaches did not result in the expected positive outcome (7). Such limitations can be overcome by multivariate deep analysis as shown in a recent sophisticated study of more than 1,400 serum proteins in 630 samples from three MS cohorts combined with clinical and radiologic disease activity that identified a MS blood biomarker panel of 20 proteins (41).

### 4.4 TCR repertoire

We found a diverse TCR repertoire in MS patients that was comparable to controls, in CSF and peripheral blood, respectively. PRS are defined as one of the top 10 sequences in MS patient’s liquor with a clone count of at least two or if detected in CSF of at least two different MS patients. We found 138 of these PRS significantly associated with MS patients. Thereof, 13 were found in McPAS database detected in CSF of up to 66.66% of MS patients and some in high frequencies (e.g., CASSQDRLTGGYTF with a maximum frequency of 1,093) but also high interpatient variability.

For a more stringent analysis, we identified MS-”predominant” CDR3.aa sequences that were either found in at least five MS patients but not in controls or that were present in both MS patients and controls but with at least a 10-fold enrichment in MS patients. We identified in total 1,846,279 private CDR3.aa sequences in our study cohort and 166,569 public sequences, i.e., shared by at least two patients. Recovery of CSF-detected clones in blood was significantly higher in MS patients, but no correlation was found with disease severity measured with EDSS at presentation, number of cells in CSF, age, or gender, in these patients. Mean = 35.7% of clones identified in MS patients’ blood were recovered in CSF, compared to mean = 37.4% in control patients, indicating that the number of shared clones in blood alone is not sufficient to discriminate MS from control patients. Similar observations apply to T-cell populations in pediatric MS patients, where abnormal ratio between regulatory and effector T cells significantly distinguishes patients from controls, but T-cell population sizes alone fail to do so (16). These facts also point toward a complex immune imbalance rather than particular cellular or humoral pathology.

### 4.5 Correlating cytokine profile with TCR repertoire

Out of 2,012,848 CDR3.aa sequences discovered in total in our study cohort, we selected 2,644 MS-”predominant” CDR3.aa sequences. The fact that 94.5% (465/492) of the correlations applied to CDR3.aa sequences found only in MS patients’ blood confirmed the specificity of our selection strategy; just 5.5% (27/492) applied to both MS and control patients. Other sequences of the MS cluster were not directly linked to a target antigen based on currently available data.

Regarding the predicted TCR targets our data largely confirm previous observations showing over-representation of clones directed against influenza, *Mycobacterium tuberculosis*, and yellow fever virus despite the limited sample size (17). These infectious disease-related targets might indicate either cross-reactivity or a role of the infectious disease as an immunological bystander mechanism that acts as an environmental risk factor by creating a pro-inflammatory situation (1). A further comprehensive in-depth analysis of the various types of immunological and genetic data is obviously needed to gain more detailed understanding of the immunopathology of MS. So far, the clear display of the Th1-biased type I immune response pattern MS already in our limited dataset indicates that future diagnostic strategies may profit from addressing immune response patterns rather than individual protein targets.

## Data Availability

The raw immunarch data were uploaded on Zenodo (DOI 10.5281/zenodo.15388205), https://zenodo.org/records/15388205.

## Supplementary material

Supplementary Figure 1 Characteristics of the study cohort. **(A)** Distribution between MS patients and control (CTRL) patients with idiopathic intracranial hypertension. Age distribution **(B)** CTRL and **(C)** MS. Gender distribution **(D)** CTRL and **(E)** MS; f, female; m, male.

Supplementary Figure 2
*(next page):* Cytokine expression levels in control (CTRL) and MS patients. Swarm plots showing **(A)** blood and **(B)** CSF cytokine levels measured in MS (violet dots) and control (CTRL) patients (green dots) as indicated. Functional groups were highlighted on top (green, chemokines; purple, growth factors; red, pro-inflammatory cytokines; blue, anti-inflammatory cytokines; orange, other cytokines). Non-detected cytokines IL-3 and IL-5 are highlighted (ND). Cytokines showing most significant differences between MS and Ctrl are highlighted in a red box (Mann-Whitney U test, adjusted p < 0.05).

Supplementary Figure 3 T cell receptor beta variable (TRBV) gene usage in blood and CSF of MS and control patients. Box plots showing the relative proportions of TRBV gene usage in blood (top) and CSF (bottom) samples from MS patients (purple boxes) compared to controls (CTRL; green boxes). Significant differences of TRBV genes between groups are marked in red (Mann-Whitney U test, *adjusted p < 0.05; ** p < 0.01).

Supplementary Figure 4 TCR CDR3.aa sequence clone count – cytokine paired correlations in control patients’ blood. Dot chart illustrating the correlation between TCR sequence (CDR3.aa log2-fold changes) and cytokine expression level in blood of control patients. We selected sequences found in at least five MS patients only (circles) or in both MS and control with at least a ten-fold enrichment for the normalized clone count analysis (diamond). Each point represents a unique significant (p < 0.05 and absolute Kendall rank correlation > 0.5) TCR sequence-cytokine pair. The color of the points indicates the strength and direction of correlation (red, positive; purple, negative), and the size of each point reflects the log2-fold enrichment of normalized clone counts in MS patients compared to controls. CDR3.aa sequences are labelled above. Red text color indicates the sequence that have a 100% match in the McPAS database.

Supplementary Figure 5 Distribution of significant TCR-cytokine correlations. Significant difference between chemokines (n = 11) and pro-inflammatory cytokines (n = 7), Mann Whitney test, p < 0.05). No significant differences for all other comparisons.

Supplementary Figure 6 Proportion of T cell receptor beta variable (TRBV) gene segments across cytokine groups. Stacked bar plot illustrating the distribution of TRBV gene segments of the significant TCR sequences/cytokine correlations in the various cytokine groups including growth factors, chemokines, anti-inflammatory, pro-inflammatory cytokines and other cytokines. Each bar represents the proportion of a specific TRBV gene segment within a group, with colors indicating different TRBV segments. Labels on the right show all TRBV segments, arranged by their highest to lowest variability. The 10 most variable TRBV segments across groups are outlined in red.

Supplementary Figure 7 Principal component analysis showing k-means clustering of estimated CD3.aa sequences. MS cluster (red) consists of 139 sequences. The general cluster (blue) includes 37 sequences found in both MS and control patients. Sequences depicted were found in the McPAS database associated with EBV or CMV.

Supplementary Table 1 PRS CDR3.aa sequence assigned to MS cluster.
