# Supplementary material for: T-cell repertoire correlates with cytokine imbalance in multiple sclerosis patients
Source: Front Immunol. 2025 Jul 1;16:1604452. doi: 10.3389/fimmu.2025.1604452 (PMC12259453; doi:10.3389/fimmu.2025.1604452)
Supplement: Supplementary Figure 1 — Characteristics of the study cohort. (A) Distribution between MS patients and control (CTRL) patients with idiopathic intracranial hypertension. Age distribution (B) CTRL and (C) MS. Gender distribution (D) CTRL and (E) MS; f, female; m, male. [file DataSheet1.docx]

**Supplementary Data**:


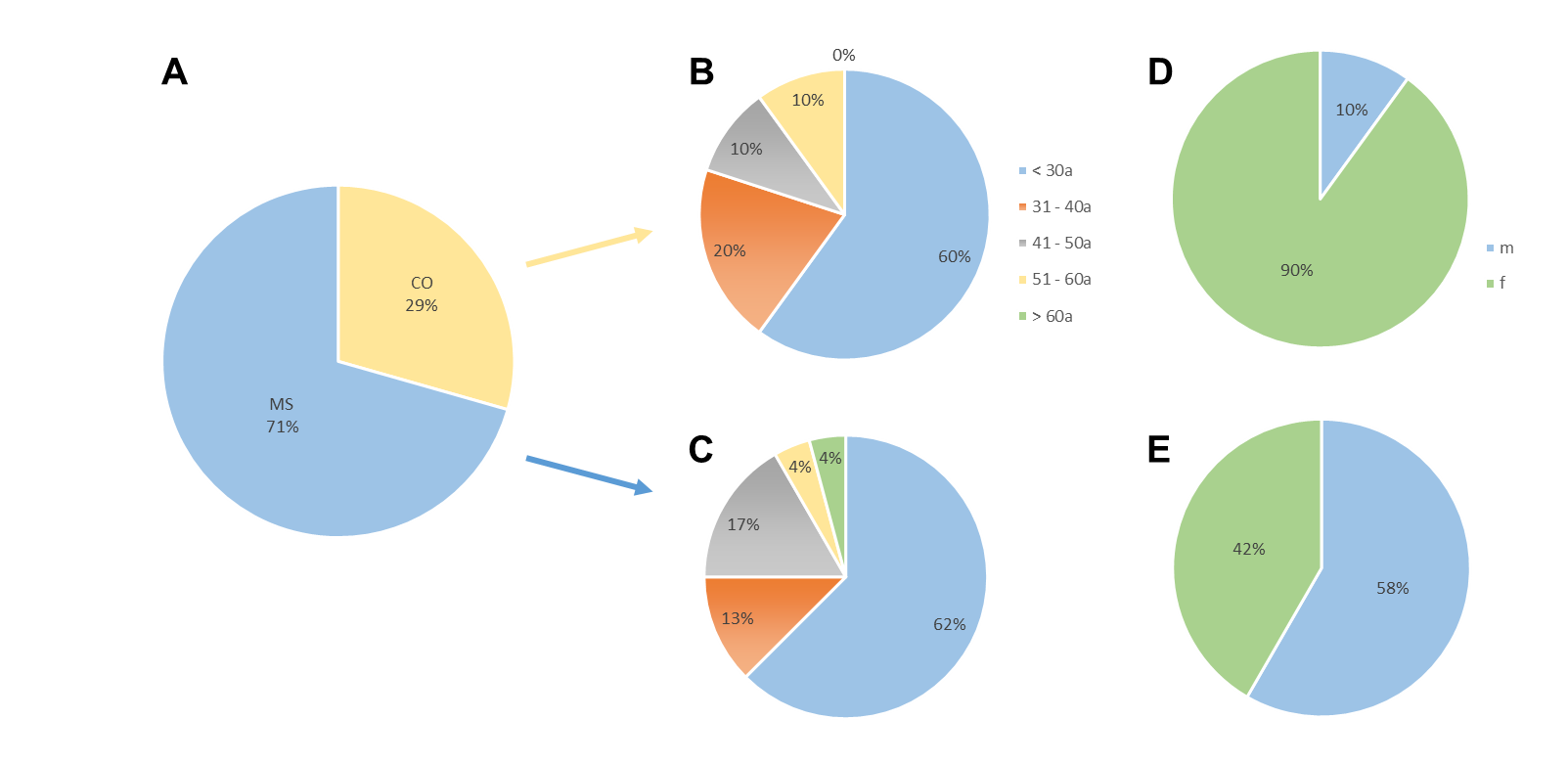
**Supplemental Figure 1: Characteristics of the study cohort.** (**A**) Distribution between MS patients and control (CTRL) patients with idiopathic intracranial hypertension. Age distribution (**B**) CTRL and (**C**) MS. Gender distribution (**D**) CTRL and (**E**) MS; f, female; m, male.

***Supplemental Figure 2 (next page):* Cytokine expression levels in control (CTRL) and MS patients.** Swarm plots showing (**A**) blood and (**B**) CSF cytokine levels measured in MS (violet dots) and control (CTRL) patients (green dots) as indicated. Functional groups were highlighted on top (green, chemokines; purple, growth factors; red, pro-inflammatory cytokines; blue, anti-inflammatory cytokines; orange, other cytokines). Non-detected cytokines IL-3 and IL-5 are highlighted (ND). Cytokines showing most significant differences between MS and Ctrl are highlighted in a red box (Mann-Whitney U test, adjusted p < 0.05).


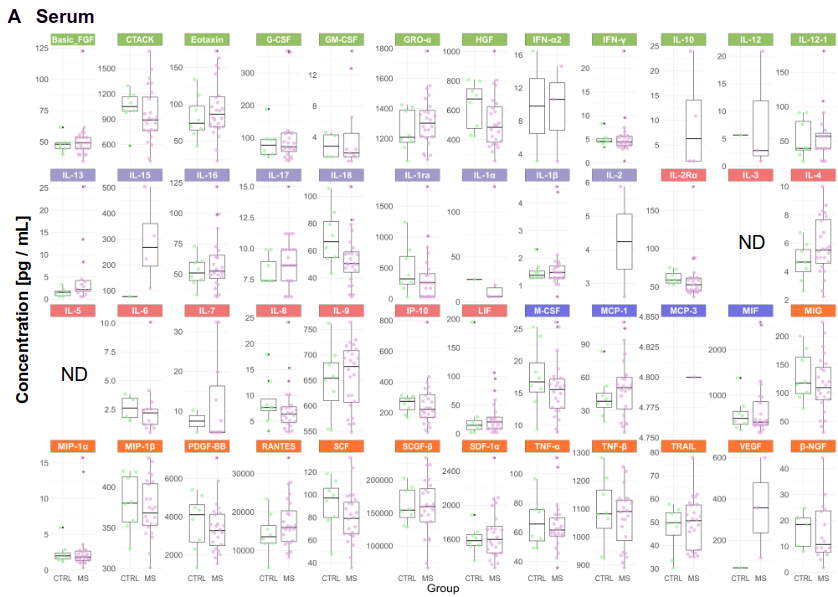

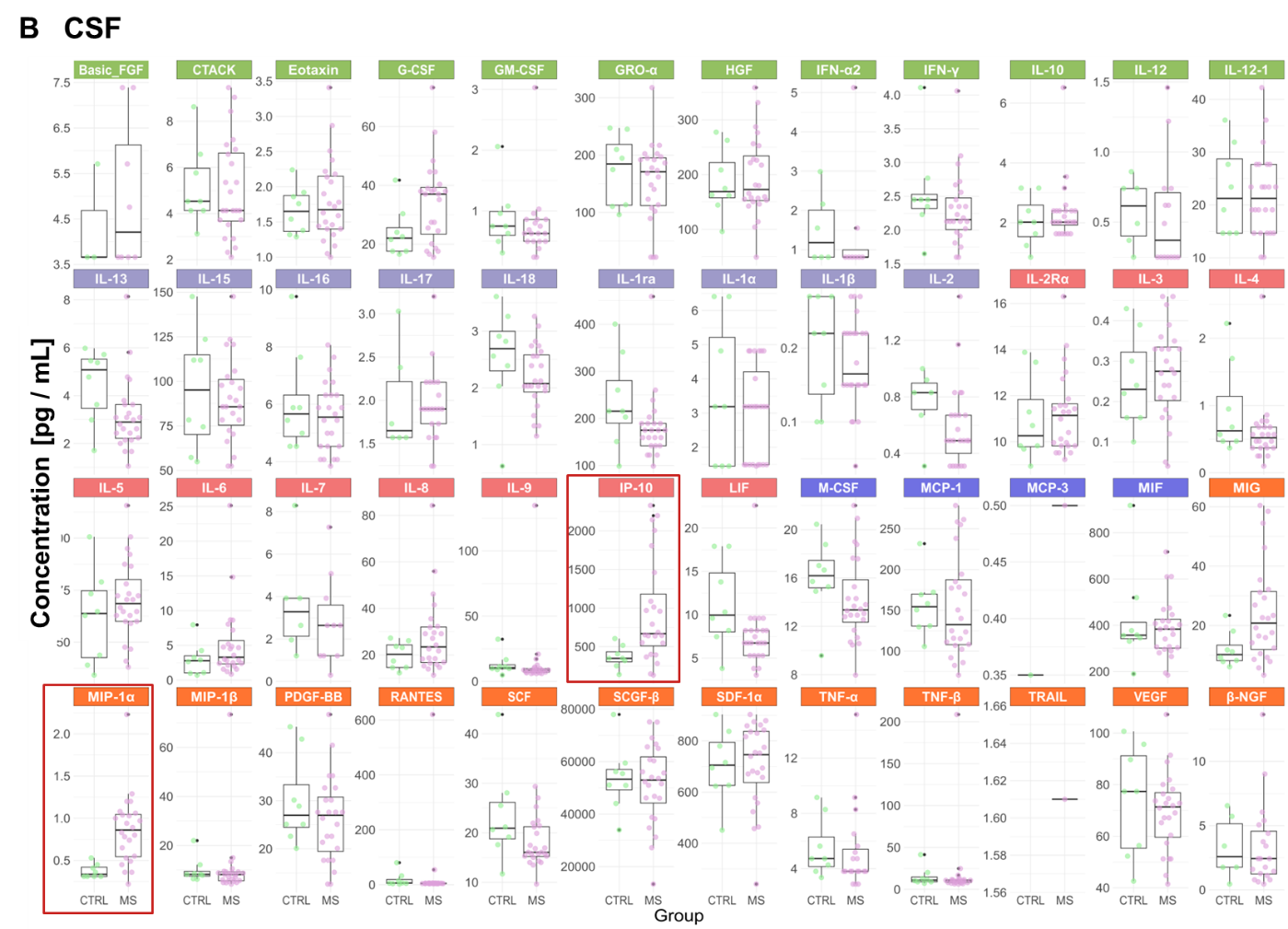


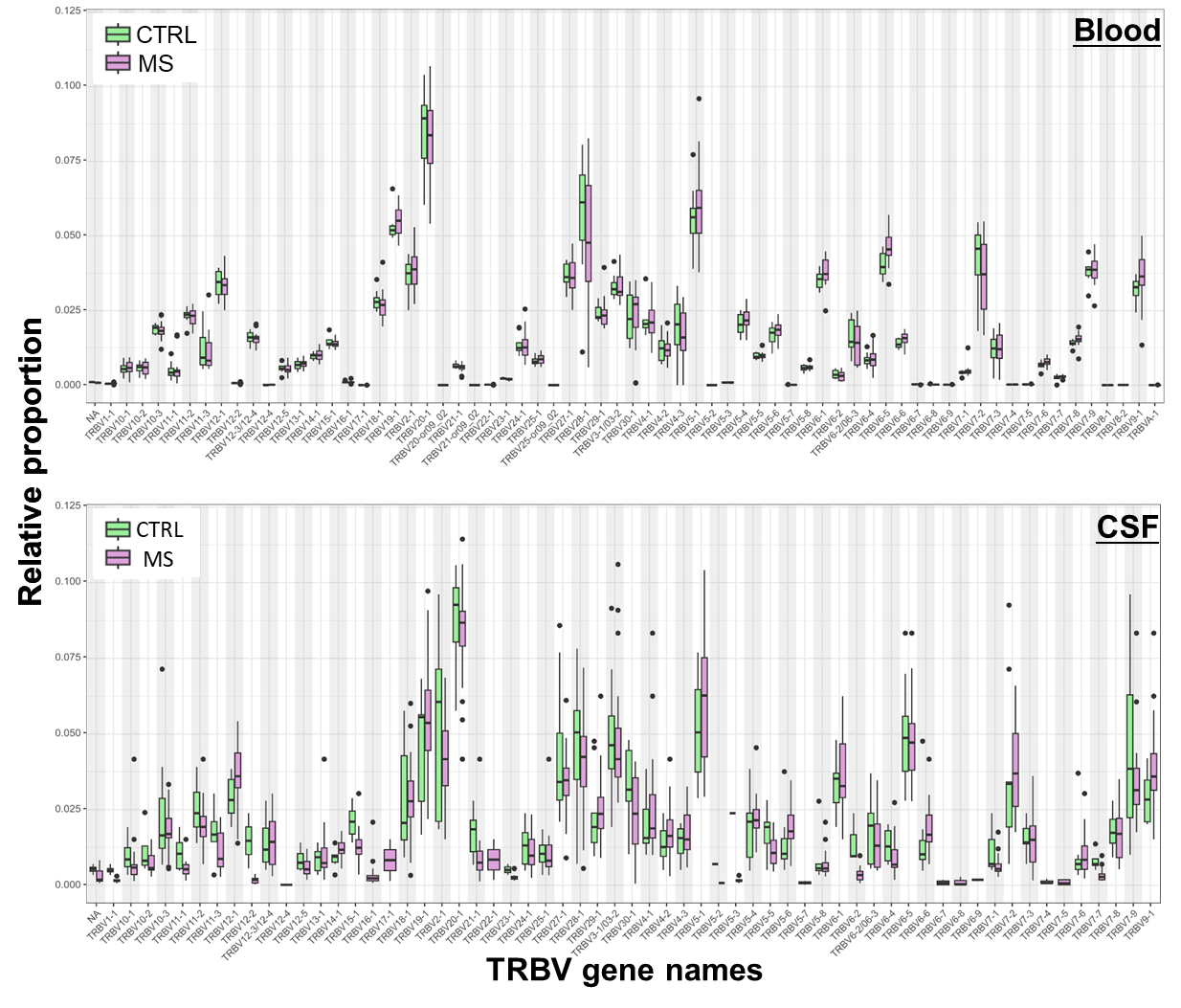


**Supplemental Figure 3: T cell receptor beta variable (TRBV) gene usage in blood and CSF of MS and control patients.** Box plots showing the relative proportions of TRBV gene usage in blood (top) and CSF (bottom) samples from MS patients (purple boxes) compared to controls (CTRL; green boxes). Significant differences of TRBV genes between groups are marked in red (Mann-Whitney U test, *adjusted p < 0.05; ** p < 0.01).


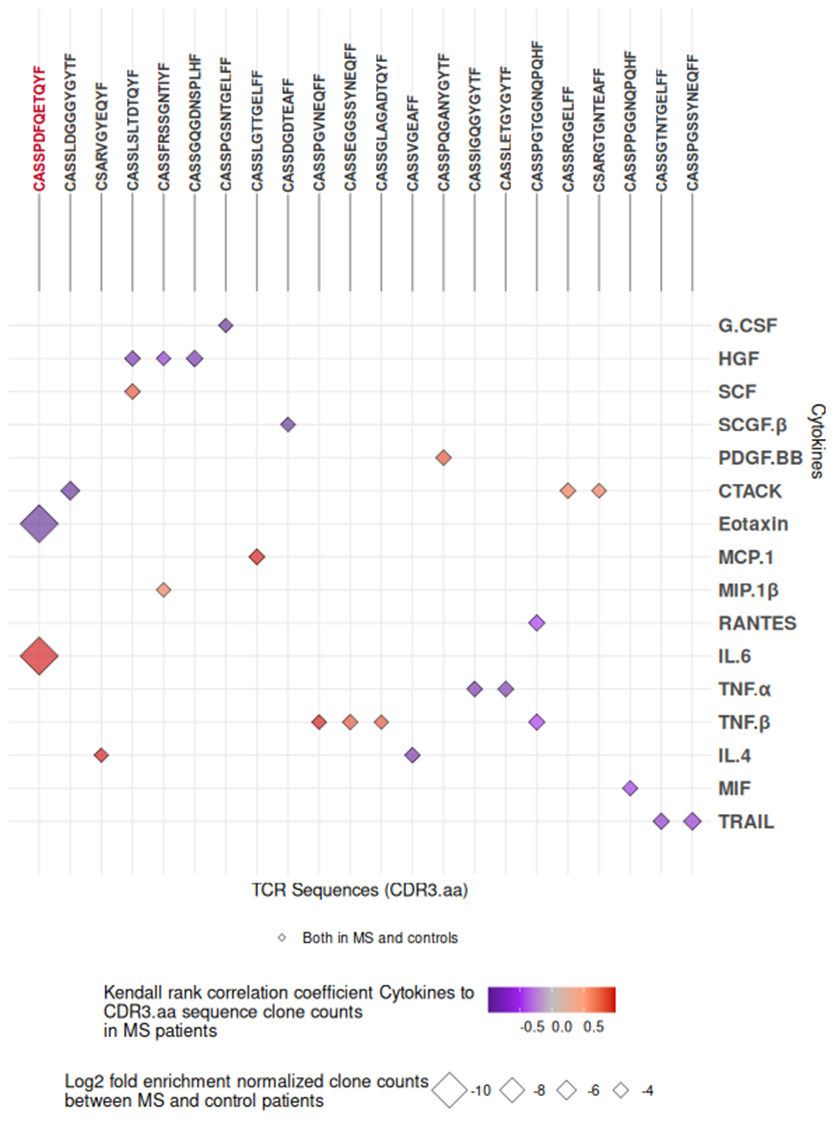


**Supplemental Figure 4: TCR CDR3.aa sequence clone count – cytokine paired correlations in control patients’ blood.** Dot chart illustrating the correlation between TCR sequence (CDR3.aa log2-fold changes) and cytokine expression level in blood of control patients. We selected sequences found in at least five MS patients only (circles) or in both MS and control with at least a ten-fold enrichment for the normalized clone count analysis (diamond). Each point represents a unique significant (p < 0.05 and absolute Kendall rank correlation > 0.5) TCR sequence-cytokine pair. The color of the points indicates the strength and direction of correlation (red, positive; purple, negative), and the size of each point reflects the log2-fold enrichment of normalized clone counts in MS patients compared to controls. CDR3.aa sequences are labelled above. Red text color indicates the sequence that have a 100% match in the McPAS database.


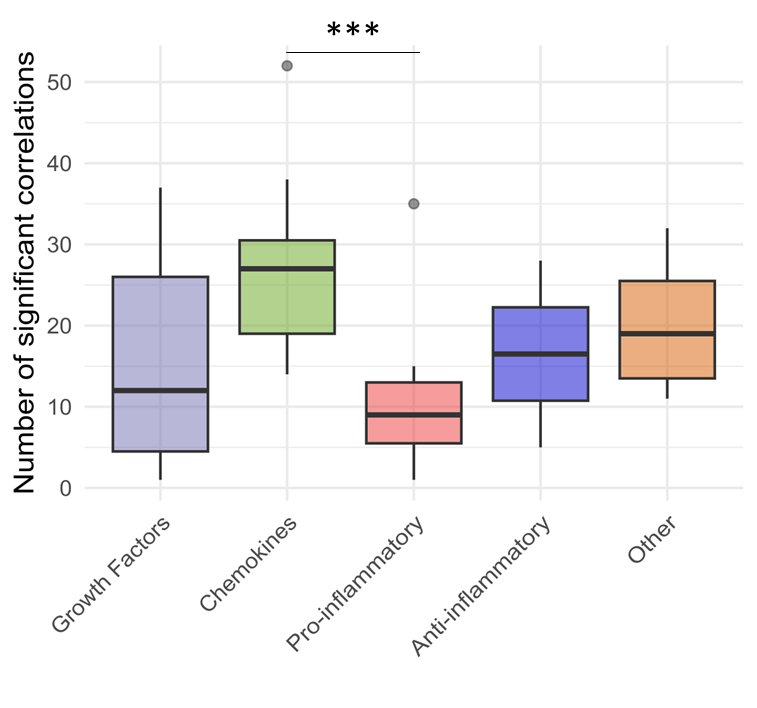
**Supplemental Figure 5:** **Distribution of significant TCR-cytokine correlations**. Significant difference between chemokines (n = 11) and pro-inflammatory cytokines (n = 7), Mann Whitney test, p < 0.05). No significant differences for all other comparisons.


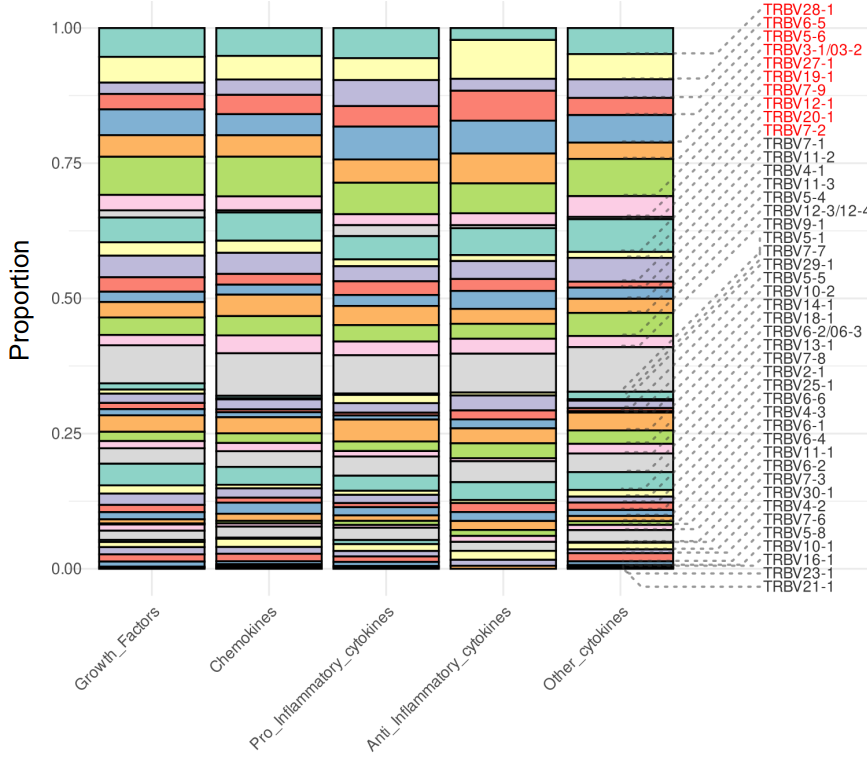


**Supplemental Figure 6:** **Proportion of T cell receptor beta variable (TRBV) gene segments across cytokine groups.** Stacked bar plot illustrating the distribution of TRBV gene segments of the significant TCR sequences / cytokine correlations in the various cytokine groups including growth factors, chemokines, anti-inflammatory, pro-inflammatory cytokines and other cytokines. Each bar represents the proportion of a specific TRBV gene segment within a group, with colors indicating different TRBV segments. Labels on the right show all TRBV segments, arranged by their highest to lowest variability. The 10 most variable TRBV segments across groups are outlined in red.


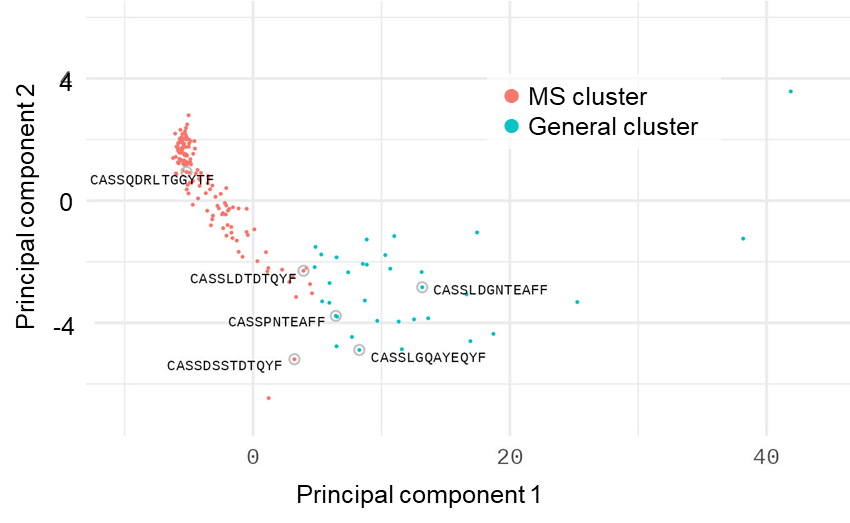


**Supplemental Figure 7: Principal component analysis showing k-means clustering of estimated CD3.aa sequences.** MS cluster (red) consists of 139 sequences. The general cluster (blue) includes 37 sequences found in both MS and control patients. Sequences depicted were found in the McPAS database associated with EBV or CMV.

**Supplemental Table 1: PRS CDR3.aa sequence assigned to MS cluster.**

| **CDR3.aa sequence in MS cluster** | | |
| --- | --- | --- |
|  | | |
| CAAEVVANQPQHF | CASSKGLAESYEQYF | CASSLTGGTYEQYF |
| CAAKTGLAERADTQYF | CASSLAAGSYNEQFF | CASSLTSAQETQYF |
| CAAREGANTGELFF | CASSLAAGTGAYEQYF | CASSLVGQGAGELFF |
| CAASEGPQHF | CASSLAGADTQYF | CASSLVGTGNEKLFF |
| CACQPGPTGGKQYF | CASSLAGGNTGELFF | CASSLVVGNTEAFF |
| CAGGGGPGETQYF | CASSLAGLSTDTQYF | CASSLYSGNTIYF |
| CAGKRHGELFF | CASSLAGRGQETQYF | CASSNYEQYF |
| CAIDPGDGELFF | CASSLDNEQFF | CASSPDGYEQYF |
| CAIRGTSSIVHEQYF | CASSLDTDTQYF | CASSPDRGNYGYTF |
| CAISDRGTEAFF | CASSLDTRNTEAFF | CASSPDSYGYTF |
| CASGDGSYEQYF | CASSLEGETQYF | CASSPGGGASYEQYF |
| CASGSPTVSYNEQFF | CASSLETDTQYF | CASSPGTSVYEQYF |
| CASKEQGPTGELFF | CASSLGAGAYNEQFF | CASSPGTYGYTF |
| CASRETGVAQPQHF | CASSLGASGSYEQYF | CASSPGYSNQPQHF |
| CASRGAGVTEAFF | CASSLGESYNEQFF | CASSPLAGGSTDTQYF |
| CASRQGNQPQHF | CASSLGGASNQPQHF | CASSPPRGETQYF |
| CASRRNTGELFF | CASSLGGLGTGELFF | CASSPPSGLTDTQYF |
| CASRTGPADTQYF | CASSLGLAGGLYEQYF | CASSPRGSSYEQYF |
| CASSARGLNGELFF | CASSLGLAGGTDTQYF | CASSPRRGTGELFF |
| CASSDRGGNSPLHF | CASSLGPNYEQYF | CASSPTGQNTEAFF |
| CASSDSSGGYNEQFF | CASSLGPRYEWQGSSNQPQHF | CASSQDRLTGGYTF |
| CASSDSSTDTQYF | CASSLGPTPYEQYF | CASSQDYEQYF |
| CASSEDRDFYNEQFF | CASSLGQAGNTIYF | CASSQGAEAFF |
| CASSEGGTEAFF | CASSLGQSSNQPQHF | CASSQGLADYNEQFF |
| CASSERETQYF | CASSLGTGMNTEAFF | CASSQGPYEQYF |
| CASSFGGADQPQHF | CASSLGVGQPQHF | CASSQGSSYEQYF |
| CASSFGGDTEAFF | CASSLQDNSPLHF | CASSQQGNYGYTF |
| CASSFGQPQHF | CASSLQGATNEKLFF | CASSQTFNTEAFF |
| CASSFGRNSPLHF | CASSLQGEQYF | CASSRDTEAFF |
| CASSFKGNTEAFF | CASSLQGSNQPQHF | CASSRGAGELFF |
| CASSGDTYEQYF | CASSLRAANSPLHF | CASSRGLADTQYF |
| CASSGGQGLNQPQHF | CASSLRDSSYEQYF | CASSRLAKNIQYF |
| CASSGGSYEQYF | CASSLRGMNTEAFF | CASSRRPTDTQYF |
| CASSIGLAGYEQYF | CASSLRGSTEAFF | CASSRTGVDQPQHF |
| CASSIGRSYEQYF | CASSLRTSGRNEQYF | CASSSGLSETQYF |
| CASSVRPGNTIYF | CASSLSGAQETQYF | CASSSGNTEAFF |
| CASSVSSGANVLTF | CASSLTGDSPLHF | CASSSGQGNEKLFF |
| CASSVTYEQYF | CASSVASSSYEQYF | CASSSGQKNTEAFF |
| CASSYGGEQYF | CASSVEVAGELFF | CASSSGYGYTF |
| CASSYGGVTEAFF | CASSVGDTQYF | CASSSNEKLFF |
| CASSYSSGGADTQYF | CASSVGGISYEQYF | CASSSRGPGELFF |
| CASSYSTDQPQHF | CASSVGGSYEQYF | CASSSRTDTQYF |
| CASTLSGANVLTF | CASSVGTGEWGDYGYTF | CASSSRVNTEAFF |
| CASTPGSGANVLTF | CASSVGTGGQPQHF | CASSTYYEQYF |
| CSARGVNTEAFF | CASSVRGDEQFF | CSARVRLAGGSGANVLTF |
| CSARSQLREQYF | CSVLEDYGYTF | CSVGGSNQPQHF |
| CSARVQQETQYF |  |  |
